# Isolation and Structure Elucidation of Three New Dolastanes from the Brown Alga *Dilophus spiralis*

**DOI:** 10.3390/md11041104

**Published:** 2013-04-02

**Authors:** Efstathia Ioannou, Constantinos Vagias, Vassilios Roussis

**Affiliations:** Department of Pharmacognosy and Chemistry of Natural Products, School of Pharmacy, University of Athens, Panepistimiopolis Zografou, Athens 15771, Greece; E-Mail: eioannou@pharm.uoa.gr

**Keywords:** *Dilophus spiralis*, dolastanes, structure elucidation

## Abstract

Three new dolastane diterpenes (**1**–**3**) and five previously reported perhydroazulenes were isolated from the organic extracts of the brown alga *Dilophus spiralis*. The structure elucidation and the assignment of the relative configurations of the isolated natural products were based on extensive analyses of their spectroscopic data, whereas the absolute configuration of metabolite **2** was determined through its chemical conversion to a previously isolated compound of known configuration.

## 1. Introduction

The family Dictyotaceae comprises cosmopolitan species of brown algae which are considered a prolific source of secondary metabolites. Representatives of the family have been the subject of numerous chemical studies over the last 50 years yielding approximately 500 new natural products. Many of these metabolites have been evaluated for and proven to possess different levels of antibacterial, antiviral, cytotoxic, antifeedant, ichthyotoxic, algicidal, and/or antifouling activities. Among these, species of the genera *Dictyota* and *Dilophus* produce mainly sesquiterpenes and diterpenes of normal biosynthesis featuring a wide range of carbon skeletons [1,2].

In the course of our ongoing research focusing on the isolation of bioactive secondary metabolites from marine organisms found along the coastlines of Greece, we initiated a thorough investigation of the chemical composition of *Dilophus spiralis* (Montagne) Hamel (syn. *ligulatus*). Previously, we described the isolation and structural characterization of five new dolastanes, one new 2,6-*cyclo*-xenicane, twenty new dolabellanes, two diterpenes featuring novel carbon skeletons, and several known compounds [3,4,5,6,7]. Herein, we report the isolation and structure elucidation of three new dolastanes (**1**–**3**) and five known perhydroazulene diterpenes.

## 2. Results and Discussion

**Figure 1:**
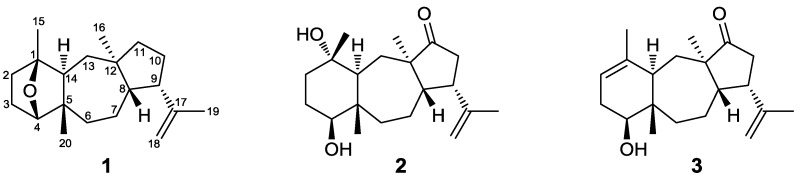
Chemical structures of compounds **1**–**3** isolated from *Dilophus spiralis*.

A series of chromatographic separations of the organic extracts of the brown alga *D. spiralis*, collected in Elafonissos island, Greece, resulted in the isolation of the new dolastanes **1**–**3** (Figure 1) and five previously reported perhydroazulenes, which were identified as dictytriene B [8], dictyoxide [9], pachydictyol A [10], isopachydictyol A [11], and dictyol E [12] by comparison of their spectroscopic and physical characteristics with those reported in the literature.

**Figure 2:**
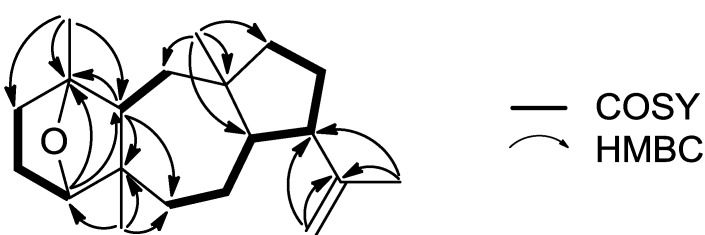
Key COSY and HMBC correlations observed for compound **1**.

Compound **1**, isolated as a colorless oil, displayed an ion peak at *m/z* 288.2459 (HRFABMS), corresponding to C_20_H_32_O and consistent with [M]^+^. The ^1^H NMR spectrum (Table 1) included signals for four singlet methyls (δ_H_ 0.86, 1.13, 1.23, and 1.64), one exomethylene group (δ_H_ 4.65 and 4.74), and an oxygenated methine (δ_H_ 3.72). The ^13^C NMR spectrum (Table 2) revealed 20 carbon signals, which corresponded to four quaternary carbon atoms, four methines, eight methylenes, and four methyls, as determined from DEPT experiments. Among them, two olefinic (δ_C_ 112.8 and 147.4) and two oxygenated (δ_C_ 84.6 and 85.5) carbons were evident. Since the carbon-carbon double bond accounted for one of the five degrees of unsaturation, the molecular structure of **1** was determined as tetracyclic. Due to the presence of two oxygenated carbons but only one oxygen atom in the molecule, in combination with the absence of an absorption band at either 1670–1750 or 3300–3500 cm^−1^ in the IR spectrum, the oxygen atom was assigned to an ether function. Comparison of these spectroscopic characteristics to those previously reported for dolastane diterpenes [3] and analysis of the correlations observed in the HMBC and COSY spectra (Figure 2) pointed to a 17-dolastene skeleton. In particular, the presence of the isopropenyl group was indicated by the long-range coupling between H_2_-18 and H_3_-19 and the HMBC correlations of C-9 and C-17 with H_2_-18 and H_3_-19, whereas the cross-peaks of H-8/H-9, H-9/H_2_-10 and H_2_-10/H_2_-11, in conjunction with the HMBC correlations of C-8, C-11 and C-12 with H_3_-16 confirmed the assignment of the five-membered ring and placed the first aliphatic methyl on C-12. The COSY correlations of H_2_-6/H_2_-7, H_2_-7/H-8 and H_2_-13/H-14, as well as the HMBC correlations of C-5 and C-6 with H-14 and H_3_-20 and of C-13 with H_3_-16 defined the seven-membered ring and fixed the position of the second aliphatic methyl on C-5. Furthermore, the HMBC correlations of C-1 and C-2 with H_3_-15, of C-4 with H_3_-20 and of C-14 with H-4 and H_3_-15, in combination with the cross-peaks of H-2/H_2_-3 and H-3β/H-4 concluded the assignment of the six-membered ring and placed the third aliphatic methyl on C-1. Finally, the correlation of C-1 with H-4 observed in the HMBC spectrum indicated that the ether bridge was positioned between carbons C-1 and C-4.

**Table 1:** ^1^H NMR data (400 MHz, CDCl_3_) of compounds **1**–**3**.

Position	1		2		3	
2	a	1.45 m	α	1.51 m		5.32 brs
	b	1.34 m	β	1.73 m		
3	α	1.83 m	α	1.71 m	α	2.24 m
	β	1.70 m	β	1.52 m	β	1.91 m
4		3.72 d (5.8)		3.19 dd (10.9, 4.0)		3.42 m
6	α	1.62 m	α	1.27 m	α	1.28 m
	β	1.27 m	β	2.24 dd (14.6, 8.4)	β	2.37 dd (14.8, 8.9)
7	a	1.68 m	α	1.67 m	α	1.77 m
	b	1.37 m	β	1.84 m	β	1.93 m
8		2.16 td (11.5, 1.6)		2.51 dt (13.8, 7.5)		2.68 ddd (13.8, 7.9, 7.5)
9		2.86 dt (11.5, 9.1)		2.77 ddd (7.8, 7.5, 2.8)		2.79 ddd (8.3, 7.9, 2.1)
10	a	1.76 m	a	2.62 dd (19.2, 2.8)	a	2.61 dd (19.2, 2.1)
	b	1.67 m	b	2.56 dd (19.2, 7.8)	b	2.56 dd (19.2, 8.3)
11	a	1.54 m				
	b	1.31 m				
13	α	1.38 m	α	2.13 dd (13.7, 2.8)	α	2.11 m
	β	1.25 m	β	1.16 m	β	1.05 t (14.0)
14		1.49 m		1.40 dd (12.6, 2.8)		2.13 m
15		1.23 s		1.07 s		1.63 s
16		0.86 s		1.03 s		1.07 s
18	a	4.74 d (2.3)	a	4.96 brs	a	4.99 brs
	b	4.65 d (2.3)	b	4.68 brs	b	4.71 brs
19		1.64 s		1.78 s		1.82 s
20		1.13 s		0.88 s		0.84 s

**Table 2:** ^13^C NMR data (50 MHz, CDCl_3_) of compounds **1**–**3**.

Position	1		2		3	
1	84.6	C	72.3	C	135.6	C
2	41.5	CH_2_	40.7	CH_2_	120.4	CH
3	24.5	CH_2_	28.4	CH_2_	32.1	CH_2_
4	85.5	CH	78.2	CH	74.9	CH
5	50.6	C	42.9	C	41.7	C
6	31.3	CH_2_	37.5	CH_2_	32.5	CH_2_
7	24.0	CH_2_	23.8	CH_2_	23.9	CH_2_
8	47.2	CH	48.5	CH	48.0	CH
9	50.1	CH	42.4	CH	42.4	CH
10	29.5	CH_2_	44.4	CH_2_	44.6	CH_2_
11	42.5	CH_2_	224.3	C	224.1	C
12	43.8	C	49.7	C	50.1	C
13	38.1	CH_2_	35.6	CH_2_	38.5	CH_2_
14	47.0	CH	51.7	CH	46.0	CH
15	18.9	CH_3_	23.0	CH_3_	23.0	CH_3_
16	21.4	CH_3_	17.2	CH_3_	17.3	CH_3_
17	147.4	C	147.0	C	147.2	C
18	112.8	CH_2_	113.3	CH_2_	113.4	CH_2_
19	23.4	CH_3_	25.8	CH_3_	26.0	CH_3_
20	27.8	CH_3_	12.0	CH_3_	9.7	CH_3_

**Figure 3:**
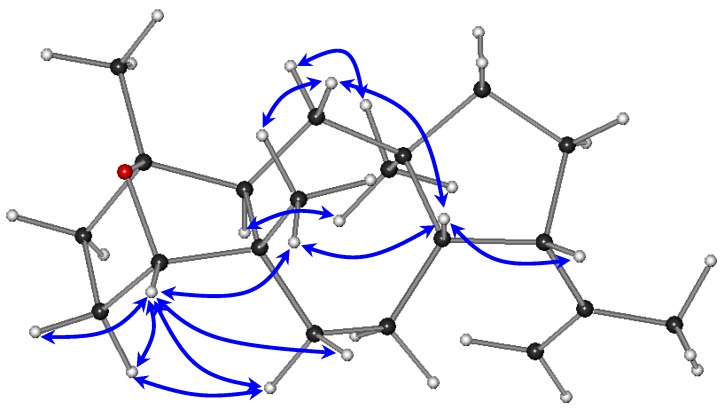
Key NOESY correlations observed for compound **1**.

The relative configurations of the stereocenters of metabolite **1** were established by analysis of the key correlations displayed in the NOESY spectrum (Figure 3). The NOE enhancements of H-8/H-9, H-8/H_3_-20, and H-14/H_3_-16 provided evidence that H-14 and H_3_-16 were cofacial, whereas H-8, H-9, and H_3_-20 were on the opposite side of the molecule, thus suggesting the *trans* fusion of the six- and seven-membered rings, as well as of the seven- and five-membered rings and determining the relative configurations of the chiral centers C-5, C-8, C-9, C-12, and C-14 as 5S*,8S*,9S*,12R*,14S*, in accordance with previously reported dolastane derivatives isolated from the same algal specimens [3]. Taking into account that the ether bridge formation between C-1 and C-4 required the *cis* orientation of the substituents at the α and α′ positions to the ether linkage, namely H-4 and H_3_-15, in conjunction with the interactions of H-4 with Η_2_-3, Η_2_-6, and Η_3_-20, as well as of Η-3α with Η-6α observed in the NOESY spectrum, the relative configurations at C-1 and C-4 were determined as 1R*,4S*. The absence of a COSY correlation between H-3α and H-4, indicating that the dihedral angle Η-3α–C-3–C-4–H-4 was approaching 90°, further supported the proposed conformation.

Compound **2**, obtained as a yellow oil, had the molecular formula C_20_H_32_O_3_, as calculated from the HRFABMS measurements and NMR data. The spectroscopic characteristics of **2** were rather similar to those of metabolite **1**. Specifically, the ^1^H NMR spectrum (Table 1), as in the case of **1**, included signals for four singlet methyls (δ_H_ 0.88, 1.03, 1.07, and 1.78), one exomethylene group (δ_H_ 4.68 and 4.96), and an oxygenated methine (δ_H_ 3.19). The ^13^C NMR spectrum (Table 2) revealed 20 carbon signals, among which one carbonyl (δ_C_ 224.3), two olefinic (δ_C_ 113.3 and 147.0), and two oxygenated (δ_C_ 72.3 and 78.2) carbons were evident. The absorption bands at 1728 and 3430 cm^−1^ observed in the IR spectrum, in conjunction with the molecular formula indicated the presence of a ketone moiety and two hydroxy groups in the molecule. The HMBC correlations of C-11 with H-8, H-9, H_2_-10, and H_3_-16 fixed the position of the ketone functionality, whereas the correlations of C-1 with H_2_-2, H-14, and H_3_-15 and C-4 with H_2_-3 and H_3_-20 placed the two hydroxy groups at C-1 and C-4. The relative configurations of the chiral centers of **2** were determined on the basis of the key correlations observed in the NOESY spectrum. In particular, the NOE interactions of H-8/H-9, H-8/H_3_-20, and H-14/H_3_-16 suggested the same relative configurations at C-5, C-8, C-9, C-12, and C-14 as in the case of **1**, while the cross peaks of H-4/H-14 and H_3_-15/H_3_-20 established the relative configurations at C-1 and C-4 as 1S*,4S*.

Compound **3** was isolated as a colorless oil. The structural elements displayed in the ^1^H and ^13^C NMR spectra of **3** closely resembled those of metabolite **2**. The ^1^H NMR spectrum (Table 1) included signals for two aliphatic and two vinylic singlet methyls (δ_H_ 0.84, 1.07, 1.63, and 1.82), one exomethylene group (δ_H_ 4.71 and 4.99), an olefinic methine (δ_H_ 5.32), and an oxygenated proton (δ_H_ 3.42), whereas the ^13^C NMR spectrum (Table 2) revealed 20 carbon signals, among which a carbonyl (δ_C_ 224.1), one oxygenated (δ_C_ 74.9), and four olefinic (δ_C_ 113.4, 120.4, 135.6, and 147.2) carbons were apparent. In agreement with the molecular formula C_20_H_30_O_2_, as deduced from the HRFABMS data, it was obvious that the difference between **2** and **3** was the absence of one hydroxy group and the formation of a second carbon-carbon double bond. The trisubstituted double bond was placed between C-1 and C-2 as indicated by the HMBC correlations of both C-4 and C-15 with H-2. The relative configurations of the stereogenic centers of **3** were established by analysis of the key NOE enhancements observed, in accordance with those of **2**, as 4S*,5S*,8S*,9S*,12R*,14R*.

Reduction of metabolite **2** according to Molander *et al.* [13] yielded the 11-deoxo derivative of **2**, identical in all respects to (1*S*,4*S*,8*S*,14*S*)-1,4-dihydroxy-17-dolastene [3]. Since the semisynthetic compound exhibited the same sign of optical rotation as the natural product previously isolated from the same algal collection [3], for which the absolute configuration was determined by application of Mosher’s method, the absolute configuration of **2** was established as depicted. The absolute configurations of metabolites **1** and **3** were not determined due to the limited available amounts, but on the basis of biogenetic considerations they are expected to be the same.

Among the new dolastanes isolated in the present study, metabolite **2**, which was obtained in adequate quantity, was evaluated for its cytotoxic activity against four human apoptosis-resistant (U373, A549, SKMEL28, OE21) and two human apoptosis-sensitive (PC3, LoVo) cancer cell lines, since previously isolated dolastanes had shown moderate cytotoxicity [3]. Furthermore, compound **2** was tested for its inhibitory effect on the hypoxia-inducible factor-1 (HIF-1). However, in both cases metabolite **2** exhibited no activity.

## 3. Experimental Section

### 3.1. General Experimental Procedures

Optical rotations were measured on a Perkin-Elmer model 341 polarimeter with a 1 dm cell. UV spectra were obtained on a Shimadzu UV-160A spectrophotometer. IR spectra were obtained on a Paragon 500 Perkin-Elmer spectrometer. NMR spectra were recorded on Bruker AC 200 and Bruker DRX 400 spectrometers. Chemical shifts are given on a δ (ppm) scale using TMS as internal standard. The 2D experiments (HSQC, HMBC, COSY, NOESY) were performed using standard Bruker pulse sequences. High resolution FAB mass spectral data were provided by the University of Notre Dame, Department of Chemistry and Biochemistry, Notre Dame, IN, USA. Low resolution EI mass spectra were measured on a Hewlett Packard 5973 mass spectrometer. Column chromatography separations were performed with Kieselgel 60 (Merck). HPLC separations were conducted using a CECIL 1100 Series liquid chromatography pump equipped with a GBC LC-1240 refractive index detector, using the following columns: (i) Econoshpere Silica 10u (Alltech, 25 cm × 10 mm) and (ii) Chiralcel OD 10 μm (Daicel Chemical Industries Ltd., Osaka, Japan, 25 cm × 10 mm). TLC were performed with Kieselgel 60 F_254_ (Merck aluminum support plates) and spots were detected after spraying with 15% H_2_SO_4_ in MeOH reagent and heating at 100 °C for 1 min. The lyophilization was carried out in a Freezone 4.5 freeze dry system (Labconco).

### 3.2. Plant Material

Specimens of *Dilophus spiralis* were collected by hand in Elafonissos island, south of Peloponnese, Greece, at a depth of 0.1–1 m, in April of 2004. A voucher specimen of the alga has been deposited at the Herbarium of the Department of Pharmacognosy and Chemistry of Natural Products, University of Athens (ATPH/MO/159).

### 3.3. Extraction and Isolation

Specimens of the freeze-dried alga (272 g) were exhaustively extracted with CH_2_Cl_2_ and subsequently with MeOH at room temperature. Evaporation of the solvents in vacuo afforded two dark green oily residues. The CH_2_Cl_2_ residue (9.2 g) was subjected to vacuum column chromatography on silica gel, using cyclohexane with increasing amounts of EtOAc, followed by EtOAc with increasing amounts of MeOH as the mobile phase, to yield fifteen fractions (A1–A15). Fraction A3 (20% EtOAc in cyclohexane, 1.17 g) was further fractionated by gravity column chromatography on silica gel, using cyclohexane with increasing amounts of EtOAc as the mobile phase, to afford twenty-one fractions (A3a–A3u). Fraction A3b (1% EtOAc in cyclohexane, 355.7 mg) was subjected to gravity column chromatography on silica gel, using cyclohexane with increasing amounts of CH_2_Cl_2_, followed by CH_2_Cl_2_ with increasing amounts of EtOAc as the mobile phase, to yield eleven fractions (A3b1–A3b11). Fraction A3b8 (100% CH_2_Cl_2_, 55.6 mg) was purified by normal phase HPLC, using cyclohexane/EtOAc (99:1) as eluant, to afford isopachydictyol A (12.4 mg) and pachydictyol A (21.4 mg). Fractions A3c (1% EtOAc in cyclohexane, 162.9 mg) and A3d (1% EtOAc in cyclohexane, 55.3 mg) were separately purified by normal phase HPLC, using *n*-hexane/EtOAc (98:2) and subsequently *n*-hexane/*i*-propanol (99.5:0.5) as eluant, to yield **1** (0.6 mg), isopachydictyol A (7.6 mg), and pachydictyol A (13.2 mg). Fraction A4 (30% EtOAc in cyclohexane, 3.58 g) was further fractionated by vacuum column chromatography on silica gel, using cyclohexane with increasing amounts of EtOAc, followed by EtOAc with increasing amounts of MeOH as the mobile phase, to afford nine fractions (A4a–A4i). Fraction A4b (10% EtOAc in cyclohexane, 46.1 mg) was purified by normal phase HPLC, using cyclohexane/EtOAc (98:2) as eluant, to yield dictyoxide (0.4 mg), isopachydictyol A (0.5 mg), and pachydictyol A (1.5 mg). Fraction A4c (20% EtOAc in cyclohexane, 812.3 mg) was subjected to gravity column chromatography on silica gel, using cyclohexane with increasing amounts of EtOAc, followed by EtOAc with increasing amounts of MeOH as the mobile phase, to afford twenty-three fractions (A4c1–A4c23). Fractions A4c2 (1% EtOAc in cyclohexane, 174.3 mg) and A4c3 (1% EtOAc in cyclohexane, 129.8 mg) were separately purified by normal phase HPLC, using *n*-hexane/EtOAc (97:3) and subsequently *n*-hexane/*i*-propanol (99.5:0.5) as eluant, to yield dictyoxide (4.1 mg), isopachydictyol A (18.4 mg), and pachydictyol A (17.6 mg). Fractions A4c15 (12% EtOAc in cyclohexane, 138.5 mg), A4c16 (20% EtOAc in cyclohexane, 13.3 mg), and A4c17 (20% EtOAc in cyclohexane, 24.7 mg) were separately purified by normal phase HPLC, using cyclohexane/EtOAc (90:10 and 92:8) as eluant, to afford **3** (0.7 mg). Fraction A10 (90% EtOAc in cyclohexane, 38.9 mg) was identified as **2**. The MeOH residue (32.8 g) was subjected to vacuum column chromatography on silica gel, using cyclohexane with increasing amounts of EtOAc, followed by EtOAc with increasing amounts of MeOH as the mobile phase, to yield fourteen fractions (B1–B14). Fraction B1 (10% EtOAc in cyclohexane, 51.0 mg) was repeatedly purified by normal phase HPLC, using *n*-hexane (100%) as eluant, to afford dictytriene B (0.9 mg). Fraction B3 (20% EtOAc in cyclohexane, 361.0 mg) was repeatedly purified by normal phase HPLC, using cyclohexane/EtOAc (90:10) as eluant, to yield dictyol E (3.0 mg). Fraction B12 (35% MeOH in EtOAc, 69.3 mg) was identified as **2**.

#### 3.3.1. (1*R*,4*S*,8*S*,14*S*)-1,4-Epoxy-17-dolastene (**1**)

Colorless oil; [α] 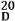 +60.0 (*c* 0.04, CHCl_3_); UV (CHCl_3_) λ_max_ (log ε) 242.5 (2.03) nm; IR (thin film) ν_max_ 2951, 2846, 1275, 908 cm^−1^; ^1^H NMR data, see Table 1; ^13^C NMR data, see Table 2; EIMS 70 eV *m/z* (rel. int. %) 288 (35), 273 (73), 255 (18), 245 (39), 227 (24), 203 (28), 187 (30), 175 (44), 161 (52), 147 (74), 135 (94), 121 (100), 107 (99), 93 (89), 79 (73), 67 (55), 55 (53); HRFABMS *m/z* 288.2459 [M]^+^ (calcd. for C_20_H_32_O, 288.2453).

#### 3.3.2. (1*S*,4*S*,8*S*,14*S*)-1,4-Dihydroxy-11-oxo-17-dolastene (**2**)

Yellow oil; [α] 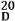 −32.0 (*c* 0.15, CHCl_3_); UV (CHCl_3_) λ_max_ (log ε) 242.0 (2.73) nm; IR (thin film) ν_max_ 3430, 2936, 1728, 1289 cm^−1^; ^1^H NMR data, see Table 1; ^13^C NMR data, see Table 2; EIMS 70 eV *m/z* (rel. int. %) 320 (17), 302 (10), 287 (18), 251 (27), 234 (35), 223 (68), 205 (83), 187 (49), 177 (41), 163 (72), 135 (86), 119 (71), 107 (79), 95 (100), 67 (80), 55 (96); HRFABMS *m/z* 319.2299 [M − H]^+^ (calcd. for C_20_H_31_O_3_, 319.2273).

#### 3.3.3. (4*S*,8*S*,14*R*)-4-Hydroxy-11-oxo-1,17-dolastadiene (**3**)

Colorless oil; [α] 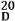 −75.8 (*c* 0.03, CHCl_3_); UV (CHCl_3_) λ_max_ (log ε) 242.5 (2.40) nm; IR (thin film) ν_max_ 3422, 2930, 1733, 1275 cm^−1^; ^1^H NMR data, see Table 1; ^13^C NMR data, see Table 2; EIMS 70 eV *m/z* (rel. int. %) 302 (11), 284 (54), 269 (19), 241 (13), 187 (27), 173 (24), 163 (37), 159 (37), 145 (40), 135 (92), 121 (65), 105 (100), 91 (73), 79 (49), 67 (31), 55 (26); HRFABMS *m/z* 303.2327 [M + H]^+^ (calcd. for C_20_H_31_O_2_, 303.2324).

### 3.4. Reduction of ***2***

Compound **2** (20.0 mg) was treated with hydrazine hydrate (100 μL) and K_2_CO_3_ (80 mg) in diethylene glycol (3 mL) and left under constant stirring at 150 °C for 1 h. Subsequently, the condenser was removed and the temperature was increased to 200 °C. After the excess of hydrazine and water had boiled off, the condenser was replaced and the temperature was maintained at 205 °C for 1.5 h. After cooling, the mixture was partitioned between CH_2_Cl_2_ and 10% HCl. The organic layer was washed again with 10% HCl and subsequently with H_2_O and saturated NaHCO_3_, dried over anhydrous MgSO_4_ and filtered. After evaporation of the organic layer *in vacuo*, the residue was purified by normal phase HPLC, using cyclohexane/EtOAc (50:50) as eluant, to afford the 11-deoxo derivative of **2** (4.7 mg).

## 4. Conclusions

A chemical investigation of the organic extracts of the brown alga *D. spiralis* led to the isolation of three new diterpenes (**1**–**3**) featuring the relatively rare dolastane skeleton isolated exclusively from marine sources and five previously reported perhydroazulenes. Their structures and relative configurations were determined on the basis of their spectroscopic data (NMR, MS, IR). The absolute configuration of **2** was determined on the basis of its chemical conversion to a dolastane of known configuration. Metabolite **2** was evaluated for but did not display noteworthy cytotoxic activity against six cancer cell lines or inhibitory effect on the hypoxia-inducible factor-1 (HIF-1). 
